# Neutrophil-to-lymphocyte ratio as a potential biomarker in predicting influenza susceptibility

**DOI:** 10.3389/fmicb.2022.1003380

**Published:** 2022-10-06

**Authors:** Guoyun Wang, Cheng Lv, Cheng Liu, Wenjun Shen

**Affiliations:** ^1^Department of Bioinformatics, Shantou University Medical College, Shantou, China; ^2^Shenzhen Qianhai Shekou Free Trade Zone Hospital, Shenzhen, China; ^3^Department of Computer Science, Shantou University, Shantou, China; ^4^Guangdong Provincial Key Laboratory of Infectious Diseases and Molecular Immunopathology, Shantou, China

**Keywords:** neutrophil-to-lymphocyte ratio, NLR, influenza susceptibility, digital cytometry, deconvolute, H3N2, H1N1

## Abstract

**Background:**

Human population exposed to influenza viruses exhibited wide variation in susceptibility. The ratio of neutrophils to lymphocytes (NLR) has been examined to be a marker of systemic inflammation. We sought to investigate the relationship between influenza susceptibility and the NLR taken before influenza virus infection.

**Methods:**

We investigated blood samples from five independent influenza challenge cohorts prior to influenza inoculation at the cellular level by using digital cytometry. We used multi-cohort gene expression analysis to compare the NLR between the symptomatic infected (SI) and asymptomatic uninfected (AU) subjects. We then used a network analysis approach to identify host factors associated with NLR and influenza susceptibility.

**Results:**

The baseline NLR was significantly higher in the SI group in both discovery and validation cohorts. The NLR achieved an AUC of 0.724 on the H3N2 data, and 0.736 on the H1N1 data in predicting influenza susceptibility. We identified four key modules that were not only significantly correlated with the baseline NLR, but also differentially expressed between the SI and AU groups. Genes within these four modules were enriched in pathways involved in B cell-mediated immune responses, cellular metabolism, cell cycle, and signal transduction, respectively.

**Conclusions:**

This study identified the NLR as a potential biomarker for predicting disease susceptibility to symptomatic influenza. An elevated NLR was detected in susceptible hosts, who may have defects in B cell-mediated immunity or impaired function in cellular metabolism, cell cycle or signal transduction. Our work can serve as a comparative model to provide insights into the COVID-19 susceptibility.

## 1. Introduction

Influenza viruses are highly contagious human respiratory pathogens that cause recurrent epidemics and occasional global pandemics. Seasonal influenza vaccines are traditionally trivalent and include components of influenza A viruses of the H1N1 and H3N2 subtypes and an influenza B virus. The pandemic influenza A(H1N1)pdm09 virus gave rise to the first influenza pandemic of the twenty-first century. The subtype H3N2 has been the most frequently occurring seasonal influenza since 1968, causing a significant threat to public health. Human population exposed to influenza viruses exhibited wide variation in susceptibility (Clohisey and Baillie, 2019). Earlier studies demonstrated that host factors, such as age, pregnancy, obesity, cardiovascular disease, and host genetics (Horby et al., 2012, 2013; Mertz et al., 2013; Patarčić et al., 2015), played a critical role in susceptibility to influenza viruses. In addition, several host factors of preexisting immune cell composition in blood have now been reported to associate with influenza susceptibility. The proportions of pre-existing CD4^+^ T cells recognizing nucleoprotein and matrix were inversely associated with total symptom scores and virus shedding of H3N2 (Wilkinson et al., 2012). The subjects having a higher proportion of pre-existing CD8^+^ T cells to conserved viral epitopes developed less severe illness after A(H1N1)pdm09 infection (Sridhar et al., 2013). Moreover, the proportion of *KLRD1*-expressing natural killer cells at baseline (i.e., prior to exposure to influenza) was lower in symptomatic shedders compared to asymptomatic nonshedders who were inoculated with H3N2 or H1N1 influenza (Bongen et al., 2018).

When there is local infection, various leukocyte populations are recruited to the infection site which is a critical early component of inflammatory responses (Luster et al., 2005; Leick et al., 2014). Neutrophils are the most abundant leukocytes in the circulation and the first to be recruited to the site of infection where they enhance local innate responses (Rosales, 2018). The innate immune system not only responses quickly to invasion by an infectious agent but also plays essential roles in activating adaptive immune responses (Clark and Kupper, 2005; Mantovani et al., 2011). While innate cells at the infection site (resident innate cells and newly recruited neutrophils) are generating antimicrobial and pro-inflammatory responses that will slow down the infection, they are also initiating steps to deliver the pathogens to lymphoid tissues where lymphocytes (T and B cells) can recognize them and generate adaptive immune responses (Schmolke and Garćıa-Sastre, 2010; Hufford et al., 2012; Chen et al., 2018). Innate immune responses and the inflammatory responses play critical roles in eliminating infections but also can be harmful when not adequately controlled. Overproduction of various normally beneficial mediators and uncontrolled local or systemic responses can cause illness and even death (Chen and Nuñez, 2010; Brandes et al., 2013). Prior studies have revealed that the host's inflammatory responses were likely to influence both the likelihood of influenza virus infection and disease severity (Hayden et al., 1998; Julkunen et al., 2000; Price et al., 2015). When the baseline levels of systemic inflammation increased, the host may be excessively susceptible to influenza virus infection (Clohisey and Baillie, 2019). The neutrophil-to-lymphocyte ratio (NLR), the ratio of the absolute neutrophil and lymphocyte counts, which can be measured during routine hematology is a simple and reliable method to evaluate the extent of systemic inflammation (Zahorec, 2001). The NLR was examined as a new prospective marker to estimate systemic inflammation and clinical outcomes in cancer patients (Templeton et al., 2014; Faria et al., 2016; Howard et al., 2019). A few studies have also reported the roles of NLR in influenza virus infection. For patients infected with avian influenza virus H7N9, the NLR taken within 24 h after admission was found to be independently associated with fatality (Zhang et al., 2019). Moreover, the NLR can be used to predict swine influenza virus infection among patients presenting with influenza like symptoms while awaiting throat swab culture and virus isolation reports (Indavarapu and Akinapelli, 2011). In patients with influenza virus infection, excessive neutrophil activation was examined to predict fatal outcome, and neutrophil-related host factors were associated with severe disease (Tang et al., 2019). Previous studies have observed a decline in lymphocyte count in patients infected with influenza virus (Chen et al., 2014; Shen et al., 2014). Both host's inflammatory responses and immune responses played essential roles in the likelihood of influenza virus infection and disease severity. The NLR which conjugates two interconnected arms of the immune system: innate immunity and adaptive immunity is an emerging biomarker of the relationships between the immune system and diseases. However, the relationship between influenza susceptibility and the baseline levels of the NLR has not been systematic investigated so far.

Digital cytometry, which quantifies cell type composition in a sample by using computational methods, allows interpretation of heterogeneous bulk blood or solid tissue transcriptomes at the cellular level. CIBERSORTx is a widely used computational method to deconvolve cell type composition and proportions (Newman et al., 2019). Existing cell type deconvolution methods normally require a signature matrix which is a collection of cell type-specific gene expression profiles. The signature matrix has been examined to determine the accuracy of deconvolution (Vallania et al., 2018). Thus, we combined CIBERSORTx with three well-defined immune signature matrices (Newman et al., 2015; Vallania et al., 2018; Monaco et al., 2019), respectively, to get reliable estimates of the NLR.

In this study, we performed a systematic investigation of the relationship between influenza susceptibility and the baseline NLR. Herein, we utilized digital cytometry to investigate heterogeneity of blood immune cell populations prior to infection. Using multi-cohort gene expression analysis, we found that the baseline NLR was significantly higher in symptomatic infected group compared to asymptomatic uninfected group. We then used a network analysis approach to identify host factors which were statistically significantly associated with the baseline NLR, and to detect several key biological pathways that may contribute to disease susceptibility to symptomatic influenza.

## 2. Results

### 2.1. Description of experimental human influenza challenge cohorts

We collected 5 human influenza virus challenge cohorts from the NCBI Gene Expression Omnibus (GEO) database. For each influenza challenge cohort, healthy adults (aged 18–45) were inoculated with A/Wisconsin/67/2005 (H3N2) or A/Brisbane/59/2007 (H1N1) influenza, and genome-wide gene expression profiles from peripheral blood collected prior to influenza challenge and the subsequent 2–7 days were assessed. All volunteers were selected based on low pre-existing immunity to the challenge virus. Subjects were classified as symptomatic or asymptomatic based on a modified Jackson score calculated from self-reported daily symptoms (Jackson et al., 1958). The infected and uninfected classification were determined by viral titers from nasopharyngeal washes using virus quantitative culture or virus quantitative PCR (Liu et al., 2016). We only considered samples from subjects whose viral titer and symptom status agree, i.e., those who were either asymptomatic and uninfected (AU) or symptomatic and infected (SI). The GSE73072 data set was profiled using Affymetrix microarrays, and it included four challenge studies which were referred to as DEE2 (H3N2), DEE5 (H3N2), DEE3 (H1N1), and DEE4 (H1N1). We utilized all samples taken before inoculation as baseline samples. The baseline samples for the DEE2 cohort were taken at −23 h post-inoculation (hpi) or immediately prior to inoculation (0 hpi), and those for the DEE5, DEE3, and DEE4 cohorts were taken at −30, −21 or 0, and −21 or 0 hpi, respectively. The baseline samples of DEE2 (H3N2) and DEE5 (H3N2) challenge studies were used as discovery cohorts, and those of the DEE3 (H1N1), DEE4 (H1N1) were used as validation cohorts. In addition, we utilized baseline samples of the GSE61754 cohort, which was profiled by Illumina microarrays as a cross-platform validation cohort. Table 1 summarizes the infection data for these included influenza challenge cohorts.

**Table 1:** Characteristics of included influenza challenge cohorts taken as baseline.

**Challenge**	**Group**	**Virus**	**Tissue**	**No. of subjects**	**No. of AU samples**	**No. of SI samples**
GSE73072(DEE2)	Discovery	H3N2	Whole blood	15	11	18
GSE73072(DEE5)	Discovery	H3N2	Whole blood	14	6	8
GSE73072(DEE3)	Validation	H1N1	Whole blood	15	12	17
GSE73072(DEE4)	Validation	H1N1	Whole blood	9	6	12
GSE61754	Validation	H3N2	Whole blood	15	8	7

All volunteers included in the challenge studies were healthy and aged 18–45 years. They were challenged with influenza A/Wisconsin/67/2005 (H3N2) or A/Brisbane/59/2007 (H1N1). All volunteers were selected based on low pre-existing immunity to the challenge virus.

### 2.2. Dissecting immune cell composition from whole blood samples

**Figure 1:**
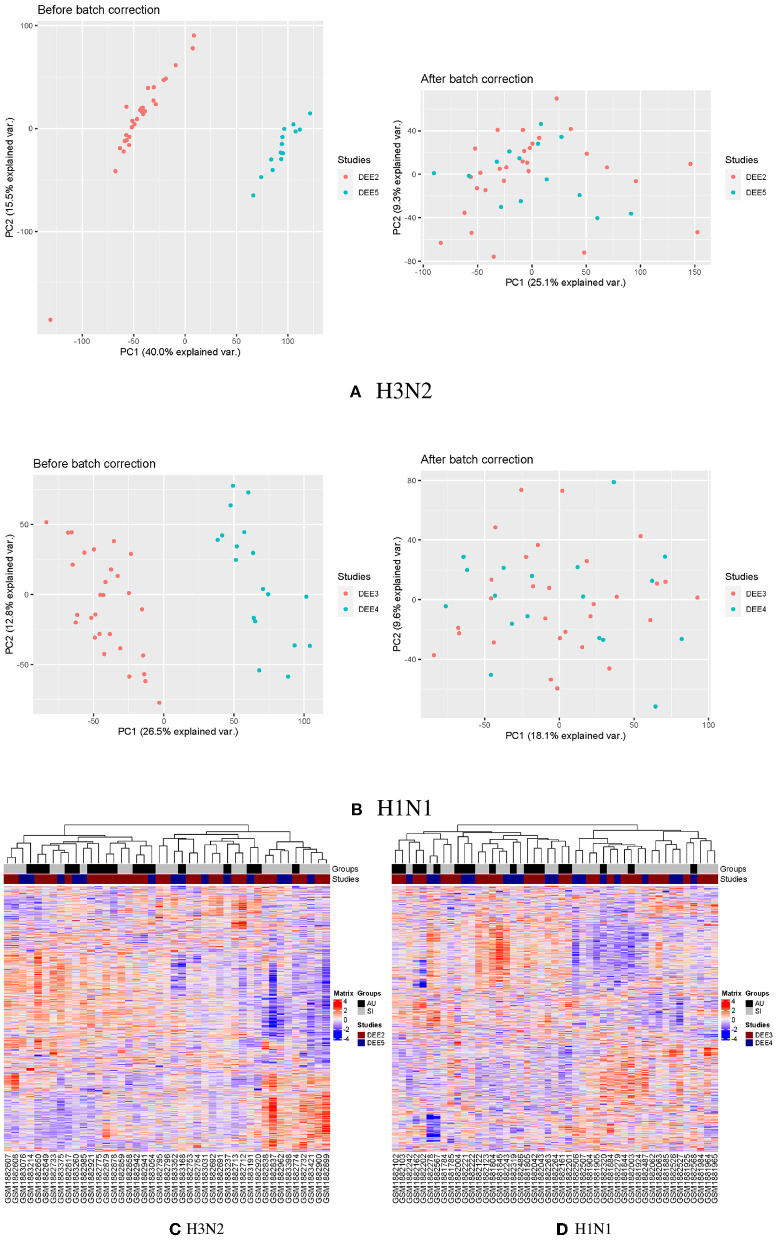
Clustering analyses on the batch corrected profiles in the GSE73072 H3N2 and H1N1 cohorts. PCA analyses of GSE73072 H3N2 **(A)** and H1N1 **(B)** cohorts before and after batch effect correction. Clustering on the samples prior to inoculation in GSE73072 H3N2 **(C)** and H1N1 **(D)** cohorts.

To explore the relationship across all samples prior to inoculation, we performed clustering analyses on the batch corrected profiles in the GSE73072 H3N2 and H1N1 cohorts, respectively (Figures 1A,B). We conducted hierarchical clustering on samples based on similarities in the top 5,000 gene expressions with the highest variance. This preliminary examination indicated that expression profiles differed between the SI and AU groups prior to inoculation (Figures 1C,D).

**Figure 2:**
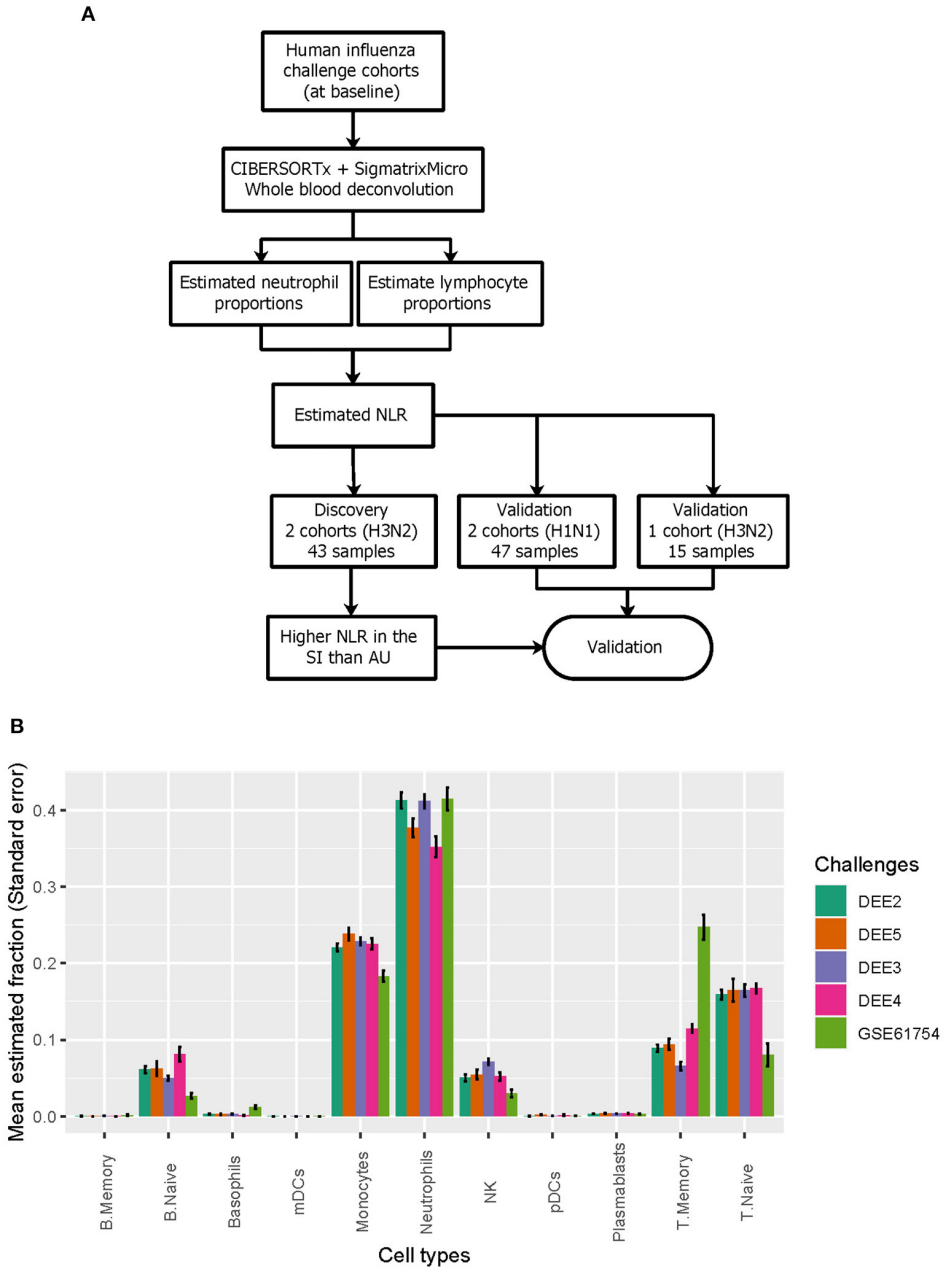
Deconvolution of immune cell populations from gene expression data. **(A)** Deconvolution of immune cell populations reveals difference in NLR between SI and AU groups. **(B)** The relative abundance of 11 immune cell populations.

We next performed digital cytometry to investigate heterogeneity of blood immune cell populations prior to inoculation (Figure 2A). To accurately dissect immune cell composition of whole blood samples from healthy subjects inoculated with live H1N1 or H3N2 influenza, we applied CIBERSORTx to expression profiles of whole blood samples by using a well-defined signature matrix named as sigmatrixMicro from a previous study (Monaco et al., 2019; see Section 4 for details). Among 11 immune cell populations dissected from whole blood samples, we observed that neutrophils were consistently dominant across five challenge cohorts, followed by T cells, monocytes, B cells, and NK cells (Figure 2B).

### 2.3. Variation in baseline NLR

**Figure 3:**
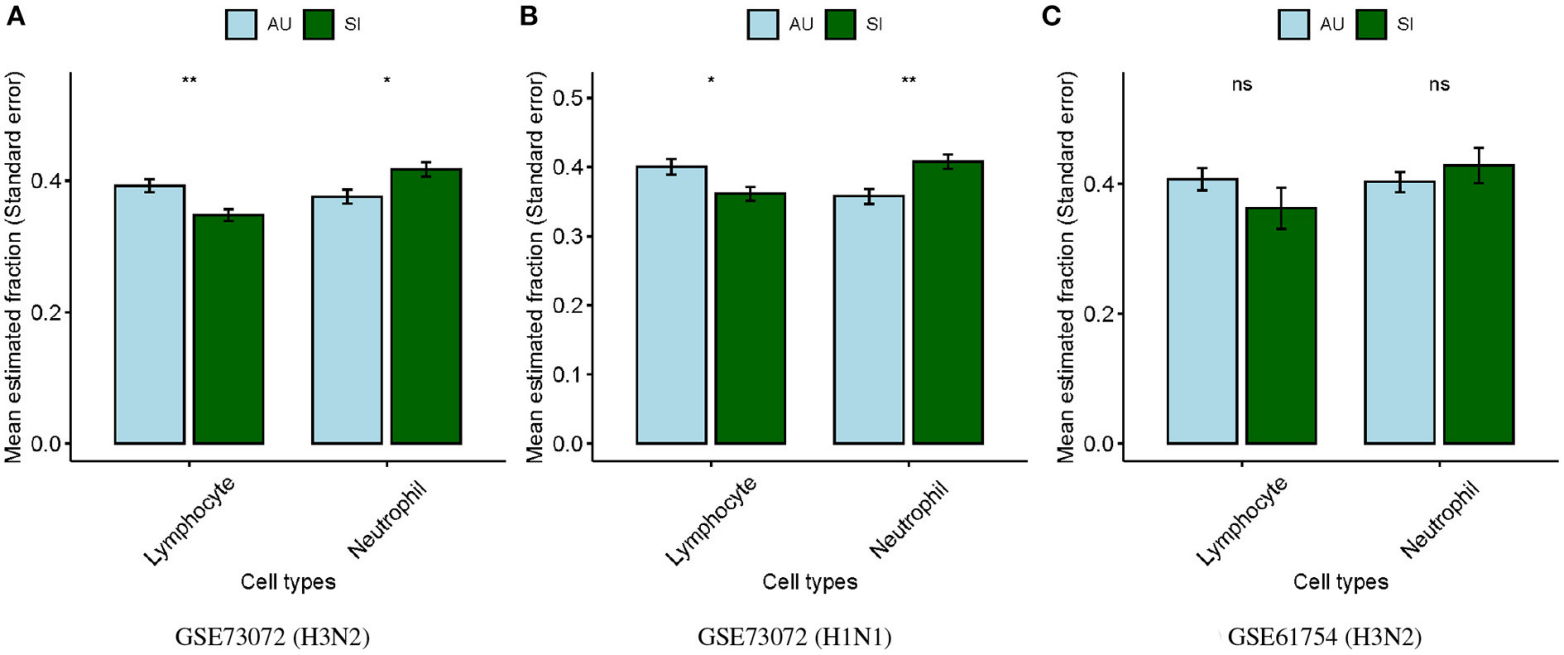
Differences in estimated lymphocyte and neutrophil proportions at baseline between SI and AU groups in the discovery **(A)** and validation cohorts **(B,C)**.

Neutrophils and lymphocytes were the two most common leukocytes in the blood. We therefore performed comparable analyses for the estimated lymphocyte and neutrophil proportions between the SI and AU groups in baseline samples. We found that proportions of lymphocytes were significantly lower (*p* < 0.01; Figure 3A) whereas proportions of neutrophils were significantly higher (*p* < 0.05; Figure 3A) at baseline in the SI group compared to the AU group in the GSE73072 cohort for H3N2 influenza. We also observed significantly lower lymphocyte (*p* < 0.05; Figure 3B) but higher neutrophil proportions (*p* < 0.01; Figure 3B) at baseline in the SI group in the GSE73072 cohort inoculated for H1N1 influenza. For the GSE61754 cohort, we observed the same trend, though these differences were not statistically significant (*p*≥0.05; Figure 3C).

**Figure 4:**
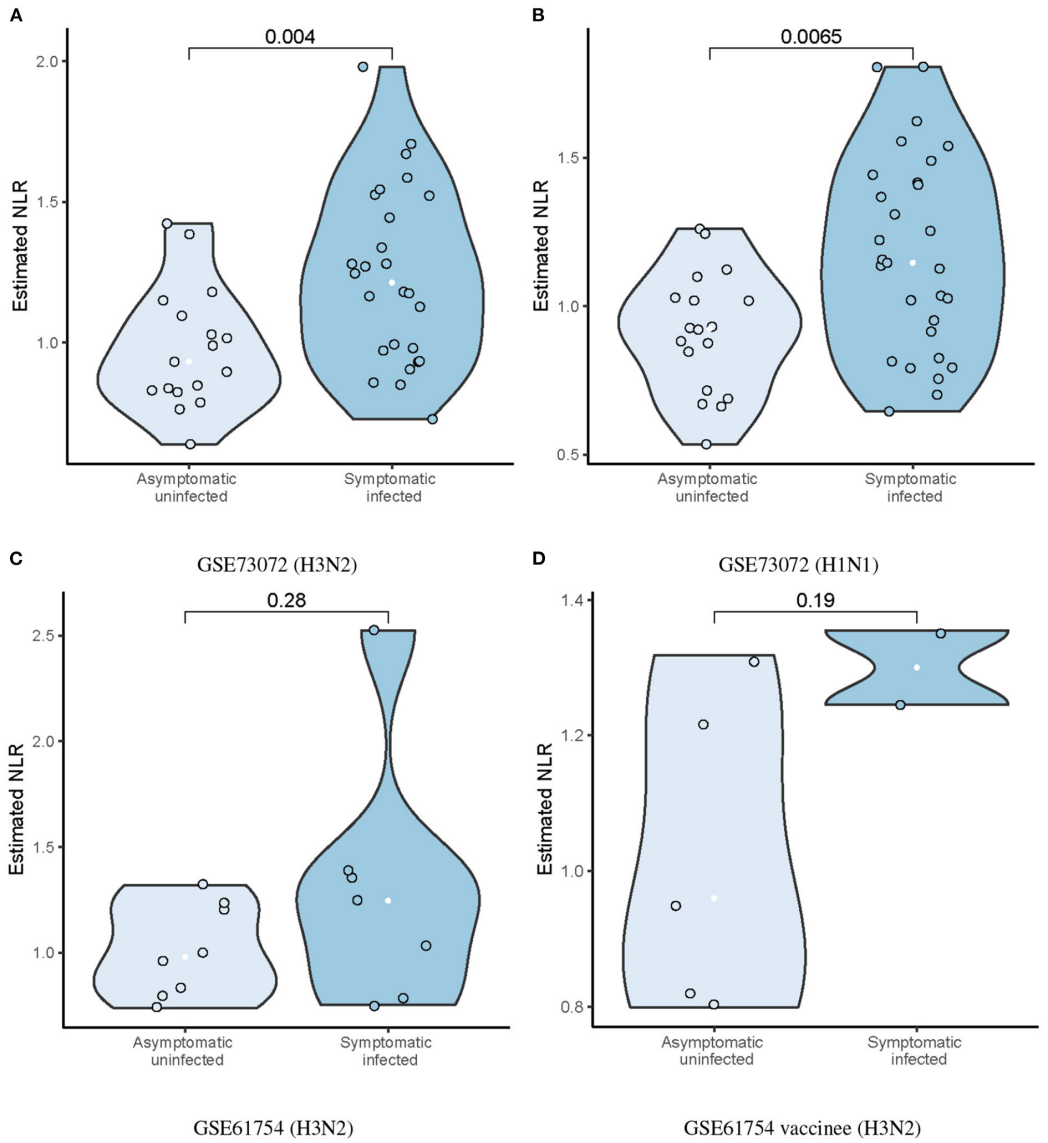
Differences in estimated baseline NLR between AU and SI groups in the discovery **(A)** and validation cohorts **(B–D)**.

The ratio of neutrophils to lymphocytes (NLR) were assessed, which was defined as the ratio of the estimated neutrophil and lymphocyte proportions. We found that the SI group had significantly higher baseline NLR than the AU group (*p* = 0.004; Figure 4A) in the discovery cohort. Higher baseline NLR in the SI group was also validated in the GSE73072 (H1N1) cohort (*p* = 0.0065; Figure 4B) and GSE61754 cohort (*p* = 0.28; Figure 4C). Although the difference in the GSE61754 cohort was not statistically significant, the baseline NLR was also higher in the SI group. In the GSE61754 cohort, 7 of 15 volunteers were vaccinated with a novel influenza vaccine MVA-NP+M1 30 days prior to influenza challenge (Davenport et al., 2015). The MVA-NP+M1 was designed to boost cross-reactive T-cell responses to antigens that were conserved across all subtypes (Lillie et al., 2012). Correspondingly, a markedly elevated memory T cell proportion was detected In the GSE61754 cohort (Figure 2B). Two of seven vaccinees developed laboratory-confirmed influenza (symptomatic infection) after challenge. We analyzed the baseline NLR between the SI and AU groups with pre-existing elevated memory T cells. The baseline NLR was still higher in the SI group, but not reached statistical significance because of the small sample size (*p* = 0.19; Figure 4D).

**Figure 5:**
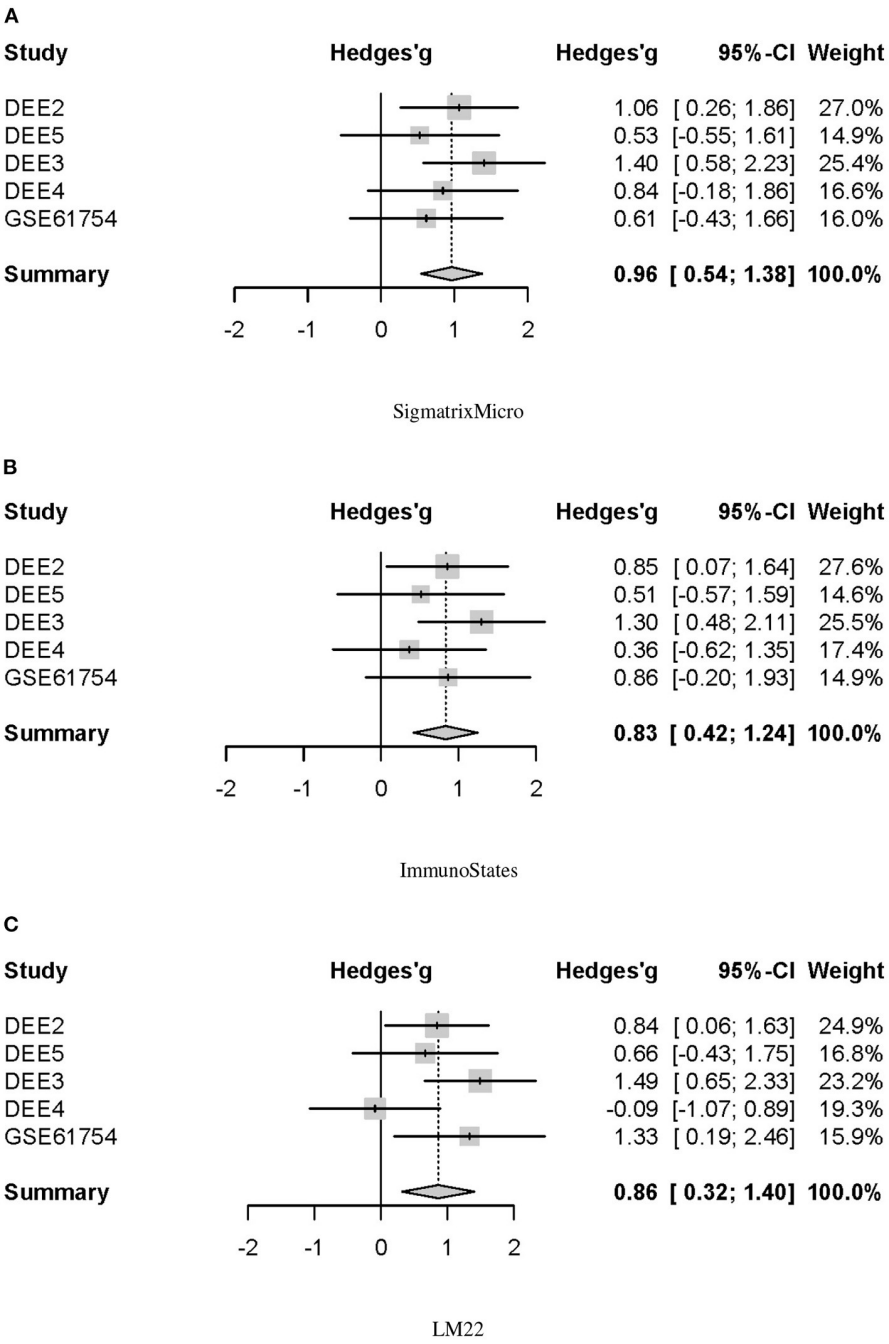
Forest plot for the baseline NLR differences between SI and AU groups in five challenge cohorts. **(A)** SigmatrixMicro. **(B)** ImmunoStates. **(C)** LM22.

We further performed a multi-cohort meta-analysis to evaluate the differences in baseline NLR between the SI and AU groups. A forest plot of estimated differences on all five challenge cohorts indicated the baseline NLR was significantly higher in the SI group (Hedges'g = 0.96, 95% CI = 0.54–1.38, *p* < 0.0001; Figure 5A). Robust *in silico* quantification of immune cell populations from peripheral blood requires a signature matrix and a deconvolution method, and the deconvolution accuracy is largely determined by a signature matrix but not a deconvolution method (Vallania et al., 2018). Therefore, two additional signature matrices of immunoStates and LM22 were tested. Differences in estimated baseline NLR were also validated by combining CIBERSORTx with immunoStates and LM22, respectively. We observed that the baseline NLR was still significantly higher in the SI group by using immunoStates (Hedges'g = 0.83, 95% CI = 0.42–1.24, *p* < 0.0001; Figure 5B) or LM22 (Hedges'g = 0.86, 95% CI = 0.32–1.40, *p* = 0.002; Figure 5C) as a signature matrix.

**Figure 6:**
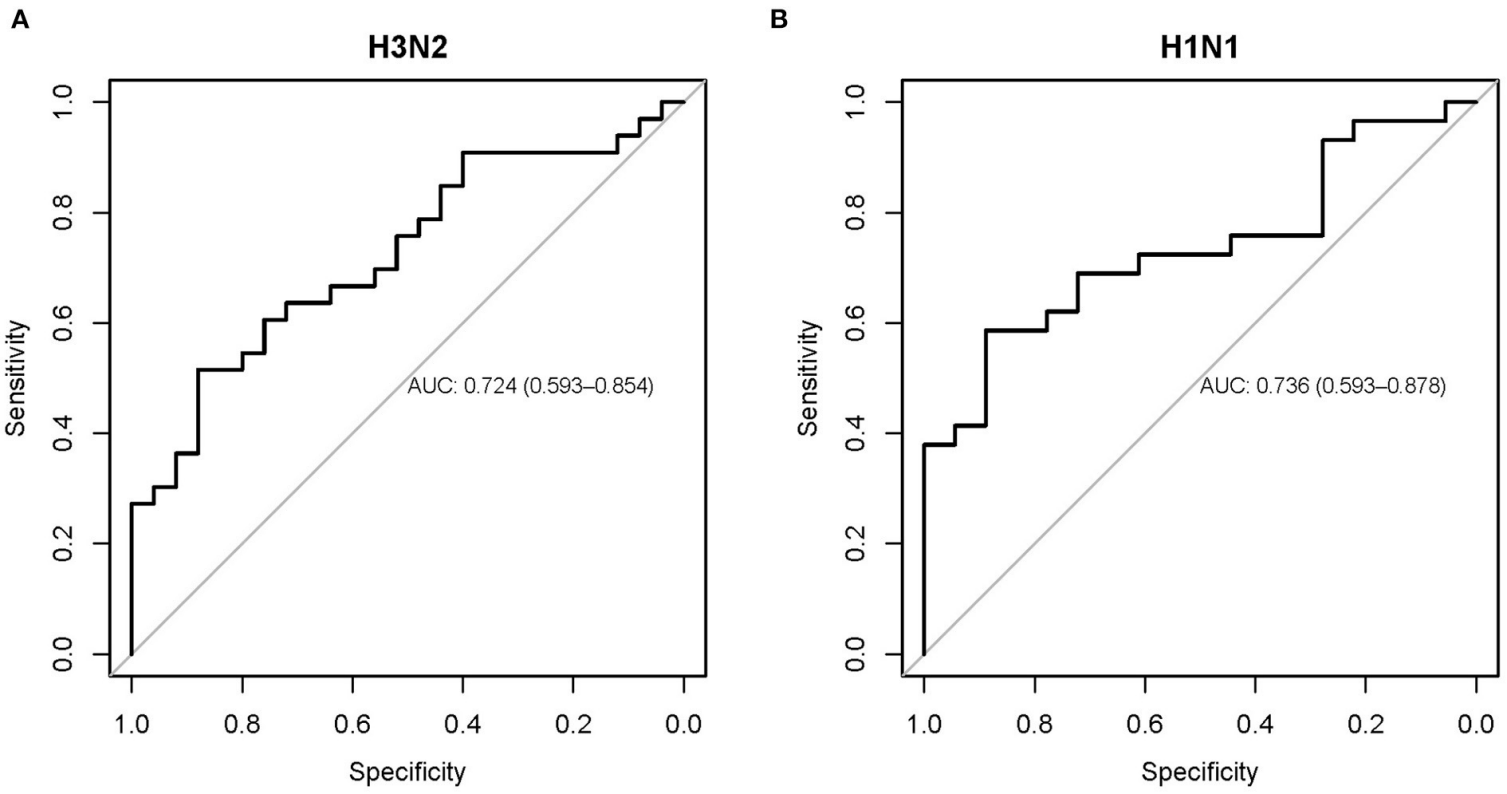
Receiver operating characteristic curve showing the performance of the baseline NLR for predicting influenza susceptibility on the H3N2 **(A)** and H1N1 **(B)** cohorts.

In addition, area under the receiver operating characteristic curve (AUC) analysis showed that the baseline NLR predicted influenza susceptibility (SI/AU) in both the challenge cohorts for H3N2 (AUC 0.724, 95% CI: 0.593–0.854; Figure 6A) and H1N1 (AUC 0.736, 95% CI: 0.593–0.878; Figure 6B) influenza.

### 2.4. NLR increases in peripheral blood after influenza virus infection

**Figure 7:**
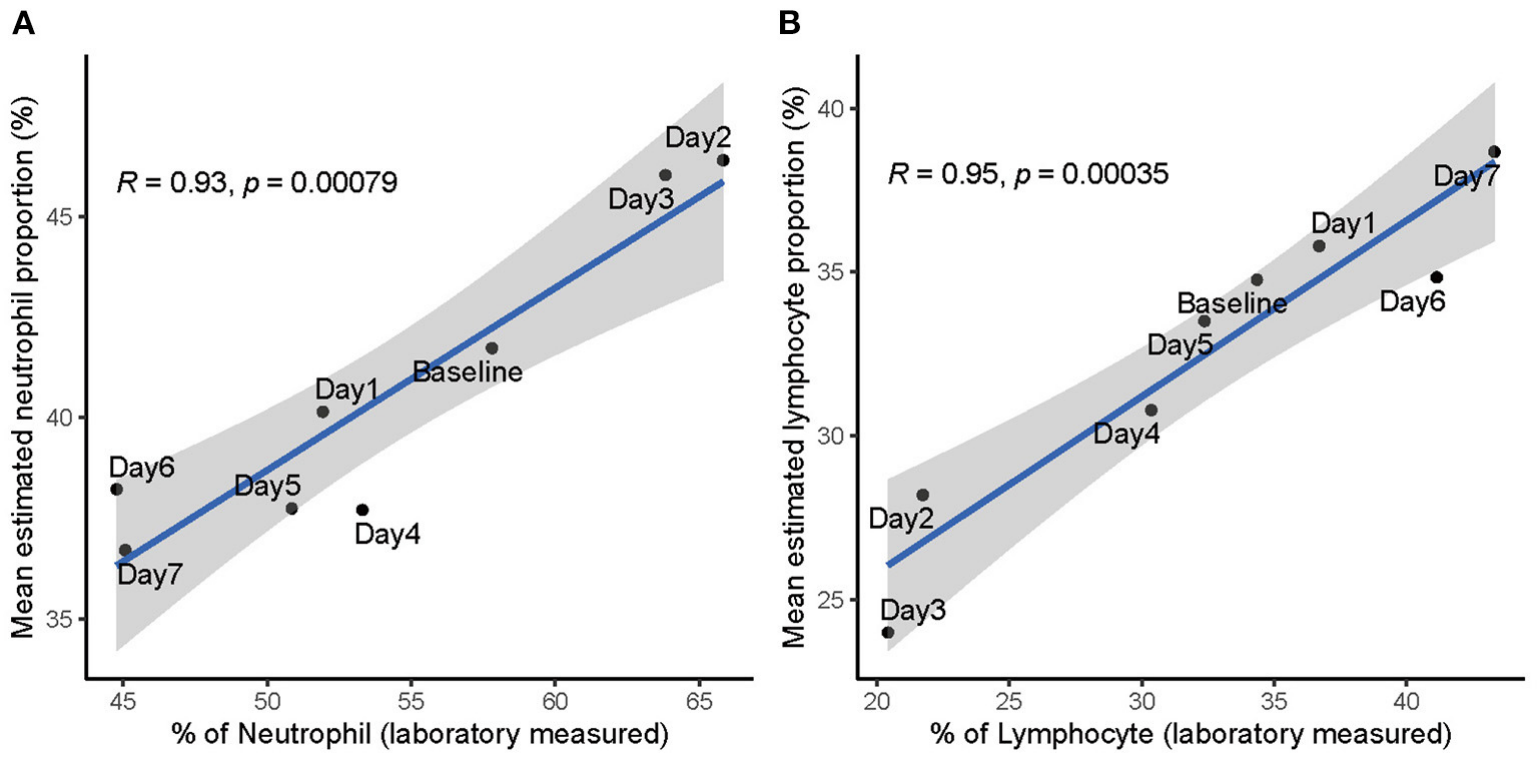
Correlation between laboratory measured and estimated proportions of neutrophil **(A)** and lymphocyte **(B)** in SI group of influenza H3N2. Data points are labeled by the day post inoculation. Baseline represents prior to inoculation and day 1 represents the day of inoculation.

To examine concordance between proportions of neutrophil/lymphocyte measured through standard laboratory workout and the deconvolution estimates, we correlated the laboratory measurements with deconvolution estimates in the SI group of influenza H3N2. We collected the laboratory measurements of neutrophil/lymphocyte proportions from Table S6 of a previous published study (Huang et al., 2011), in which they involved the same volunteers included in the GSE73072 H3N2 cohort. The laboratory measurements of neutrophil/lymphocyte proportions were obtained daily from day 1 to 7 including baseline (prior to inoculation). In the SI group of GSE73072 H3N2 cohort, the mean estimated proportions of neutrophil/lymphocyte were strongly positively correlated with the laboratory measurements (Neutrophil: *R* = 0.93, *p* = 0.00079, Figure 7A; Lymphocyte: *R* = 0.95, *p* = 0.00035, Figure 7B). These data further validate the deconvolution approach can correctly estimate the proportions of neutrophil and lymphocyte, as well as the NLR.

**Figure 8:**
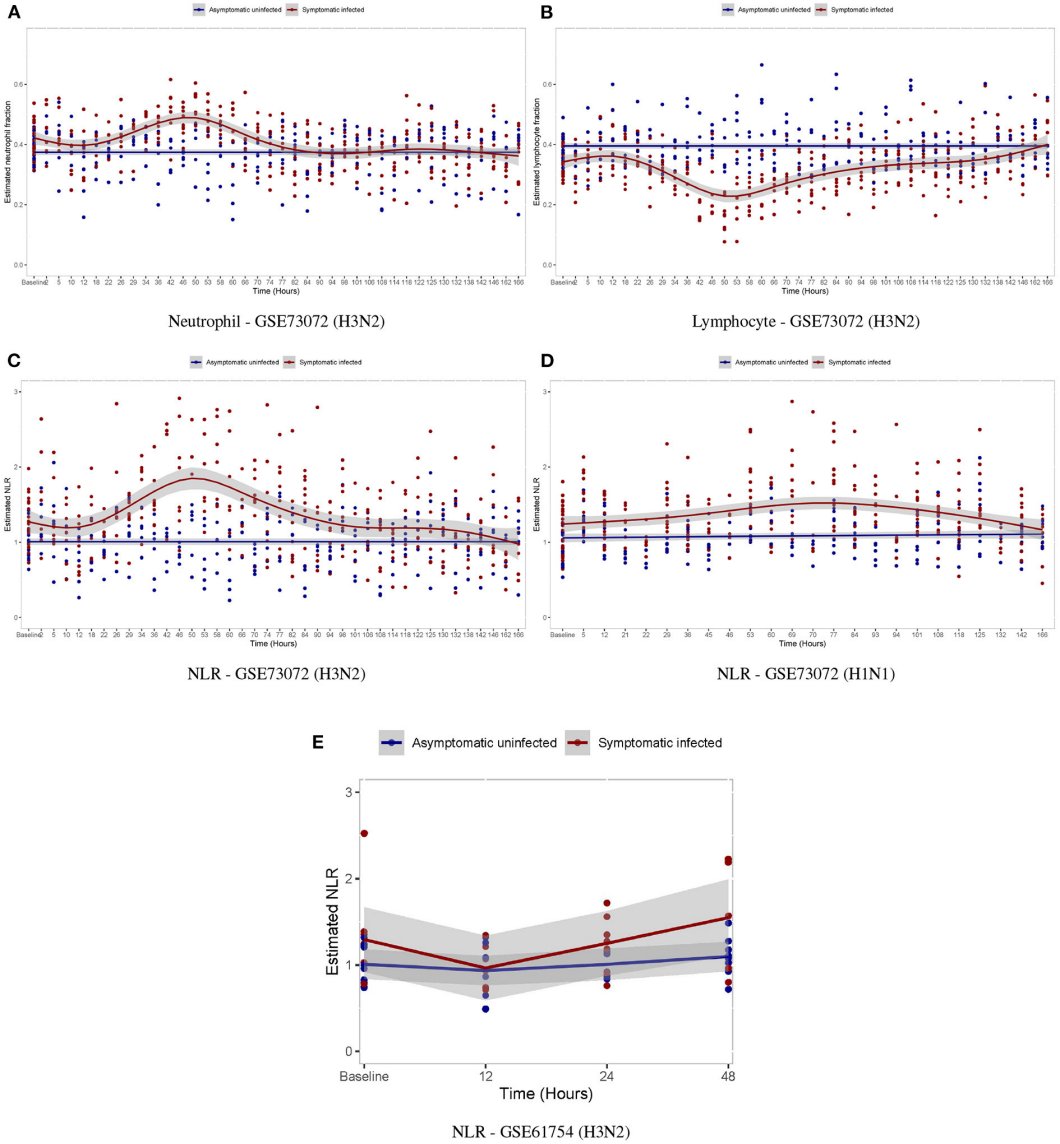
Temporal changes in white blood cell proportions among volunteers after inoculation with influenza virus. **(A,B)** Temporal changes in neutrophil/lymphocyte proportions in the GSE73072 H3N2 cohort. **(C–E)** Temporal changes in NLR among individuals in three cohorts.

We further investigated the temporal alterations of the neutrophil/lymphocyte proportions by influenza virus infection. In the GSE73072 H3N2 cohort, we observed that subjects in the AU group demonstrated no significant changes in the neutrophil/lymphocyte proportions at any time post-inoculation (Figures 8A,B). However, subjects in the SI group underwent a slightly drop in the neutrophil proportion by 12 hpi in the early stage and then a significantly rise by day 2 post-inoculation, while experience a concomitant rise and fall in the lymphocyte proportions (Figures 8A,B). The temporal changes in the neutrophil/lymphocyte proportions we estimated using digital cytometry method were consistent with the changes detected using white blood cell counts in laboratory measurements (Figures 7A,B) (Douglas et al., 1966; Huang et al., 2011; McClain et al., 2013). Our findings parallel the observation of a relative lymphopenia/neutrophilia in influenza virus infection, caused by a leukocyte redistribution between blood, lymph nodes, and tissues. This redistribution is usually transient and profound changes appear on day 2 post-infection (Music et al., 2016).

For the temporal alterations of the NLR by influenza virus infection, in both the discovery (Figure 8C) and validation cohorts (Figures 8D,E), we observed that subjects in the AU group demonstrated no significant changes at any time post-inoculation, while those in the SI group underwent a significantly rise by days 2–3 post-inoculation and then gradually returned to baseline by day 7 post-inoculation as symptoms resolved.

**Figure 9:**
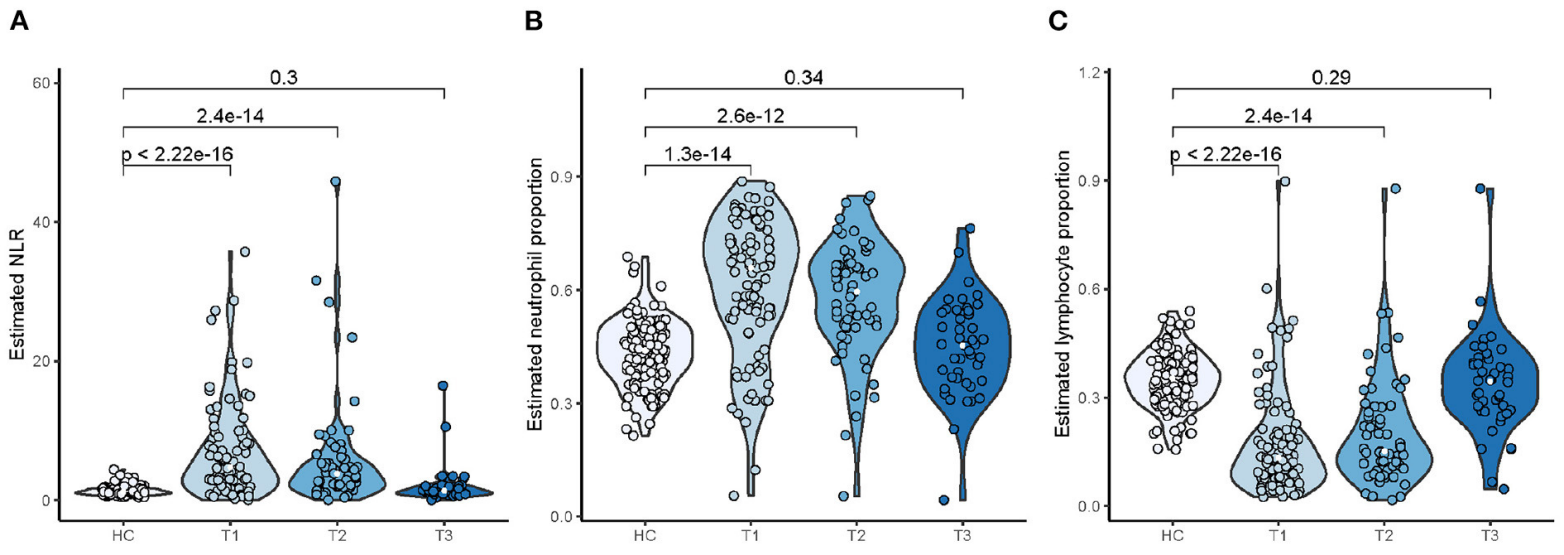
Temporal changes in NLR **(A)**, neutrophil **(B)**, and lymphocyte **(C)** proportions among patients hospitalized with influenza.

In the GSE111368 data set, samples of 94 adult patients hospitalized with A(H1N1)pdm09 influenza virus infection were collected at three time points T1 (recruitment), T2 (~2 days after T1), and T3 (at least 4 weeks after T1) covering the periods of influenza illness and clinical recovery. We observed that infected patients developed a significant increase in the NLR (Figure 9A) and neutrophil proportion (Figure 9B) compared to healthy control subjects (HC) during the period of influenza illness (T1 and T2), whereas there was no significant difference in the NLR compared to HC once the patients had clinically recovered (T3). An opposite alteration in lymphocyte proportion was detected (Figure 9C). The temporal changes in the neutrophil/lymphocyte proportions and the NLR among patients hospitalized with influenza were consistent with the changes detected in the influenza challenge cohorts.

### 2.5. Network analysis identified four disease modules associated with baseline NLR

**Figure 10:**
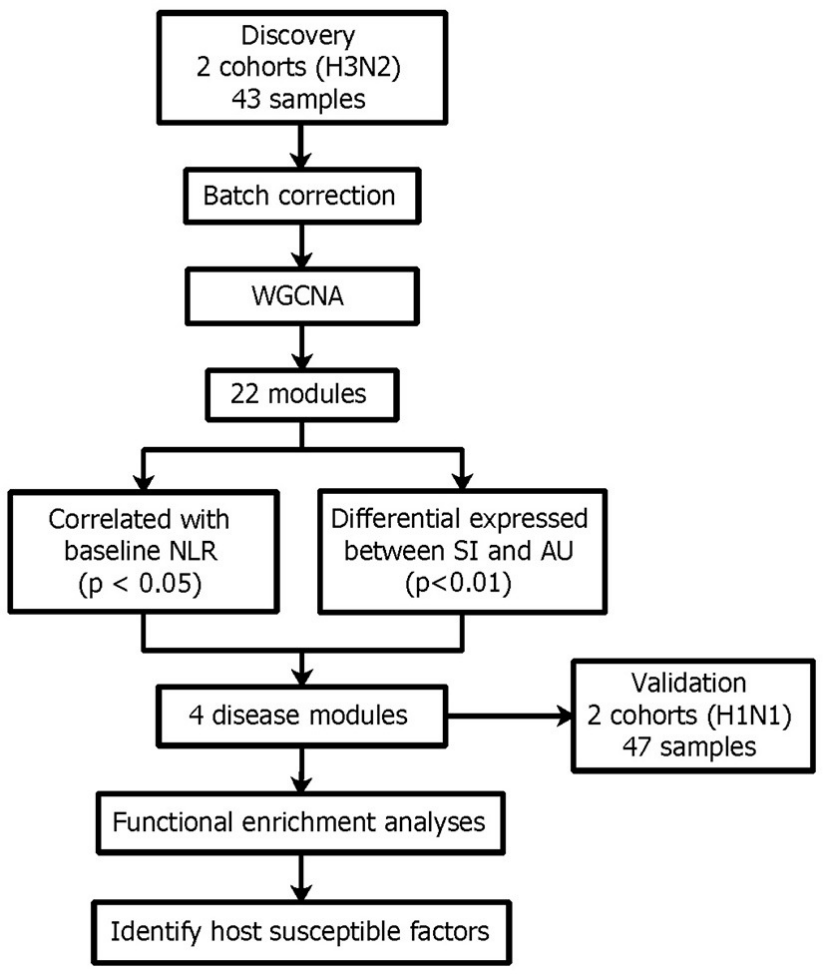
Network analysis reveals four disease modules associated with the baseline NLR and also differentially expressed between the SI and AU groups.

To investigate disease modules associated with the baseline NLR, we performed gene co-expression network analysis in the discovery cohort using WGCNA (Zhang and Horvath, 2005; Figure 10). WGCNA constructs a network based on the pairwise correlations between gene expression profiles. It has been demonstrated that batch effects can lead to false correlations between gene expression profiles, thus introduced false edge connections or lose true edge connections (Parsana et al., 2019). Principal component analysis (PCA) revealed that both the discovery and validation cohorts included two sample batches (Figures 1A,B), therefore we regressed out the batch effects using linear models and constructed co-expression networks using batch corrected profiles (see Section 4 for details).

**Figure 11:**
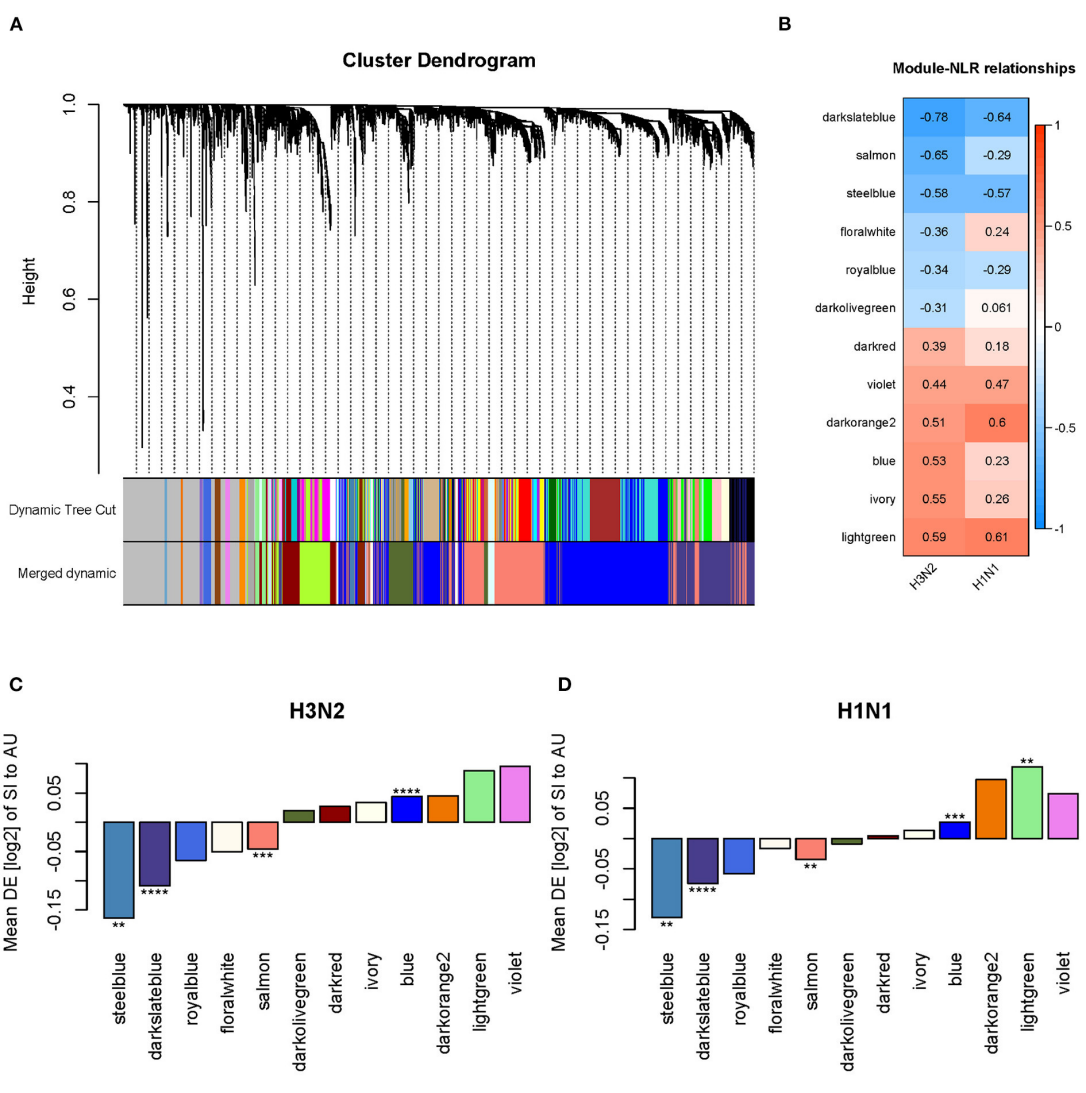
Network analysis identified four disease modules associated with the NLR. **(A)** Gene dendrogram and co-expressed modules obtained by WGCNA. **(B)** Statistically significant modules correlated with the NLR. **(C,D)** Differential expression (DE) of SI group compared to AU group in each module in the discovery and validation cohorts (*p < 0.05, **p < 0.01, ***p < 0.001, ****p < 0.0001).

The WGCNA method found clusters (modules) of genes with highly correlated expression profiles and interconnectivity across samples. Using WGCNA, we build a gene dendrogram by using the topological overlap measure (TOM) as a proximity measure. We identified modules using dynamic tree cut approach and those closely related modules whose correlations of module eigengenes larger than 0.75 were merged. We then detected 22 distinct gene modules from the dendrogram (Figure 11A). To identify modules associated with the NLR, we calculated Pearson's correlation coefficient between the module eigengenes and the NLR. Of these 22 identified modules, 12 modules were statistically significant (*p* < 0.05) when correlated with the NLR for H3N2 (Figure 11B). In the GSE73072 cohort for H1N1, we observed the same direction of the correlation value in 10 of these 12 modules (Figure 11B). Furthermore, significant differential expressions between the SI and AU groups were detected in 4 of these 12 modules (Figure 11C). Three modules (steelblue, darkslateblue, salmon) were negatively correlated with the NLR and significantly down-regulated in the SI group, while the blue module was positively correlated with the NLR and significantly up-regulated in the SI group (Figure 11C). All four modules also shown significant difference in the validation cohort (GSE73072 H1N1; Figure 11D). Hence, these four modules were identified as significant disease modules associated with the NLR: module steelblue with 70 genes, module darkslateblue with 1,296 genes, module salmon with 1,526 genes, and module blue with 2,388 genes in total.

### 2.6. Functional enrichment analysis in four disease modules associated with baseline NLR

**Figure 12:**
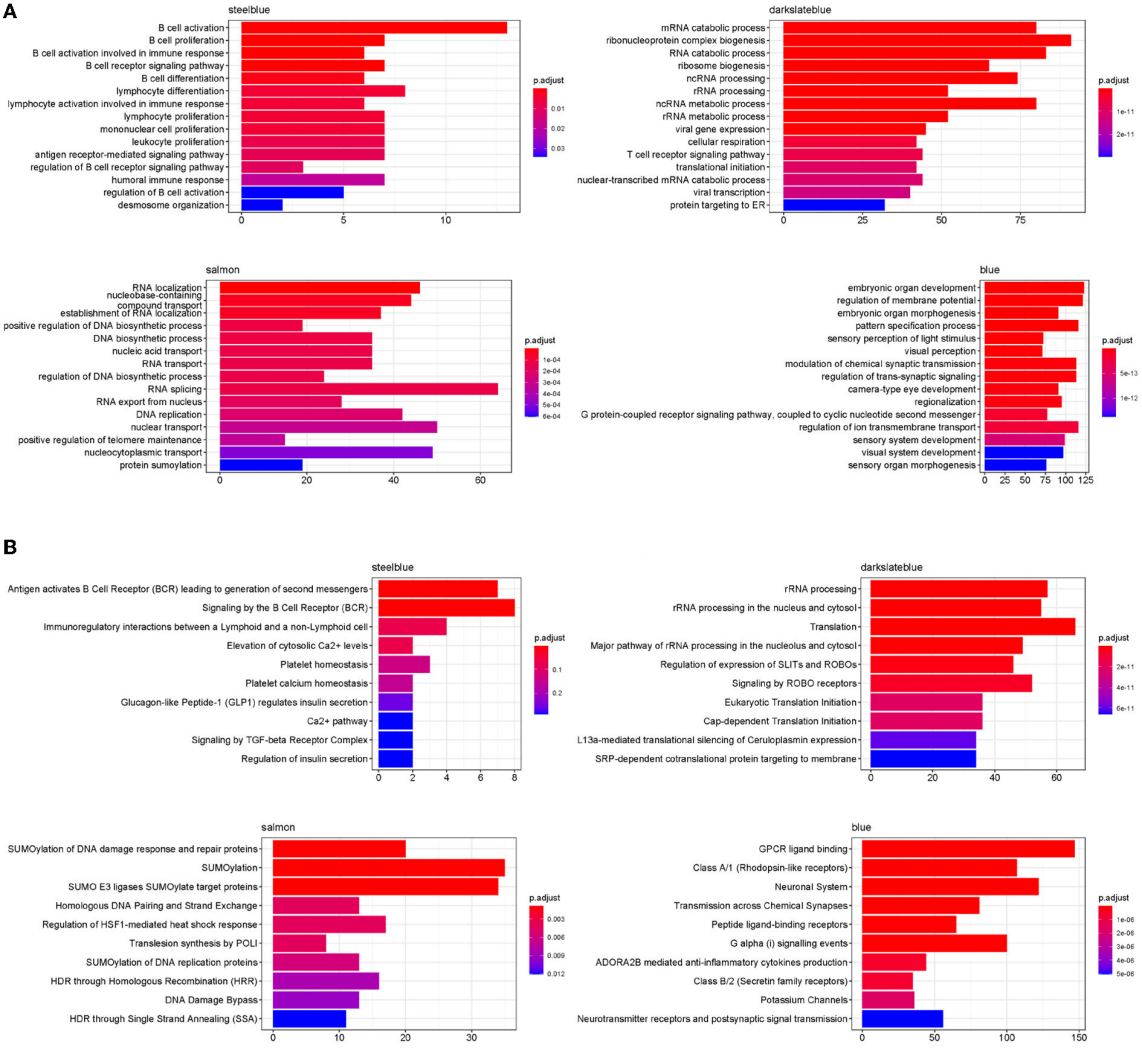
Functional enriched terms in four disease modules. **(A)** Top 15 enriched GO terms for biological processes in four disease modules. **(B)** Top 10 enrichment of Reactome ontologies in four disease modules.

To investigate the biological functions of the four disease modules, the enrichment of Gene Ontology (GO) Biological Process and Reactome ontologies in each module were analyzed and the top terms of each category are shown in Figures 12A,B. The GO enrichment results revealed that the steelblue module was significantly enriched in B cell activation, proliferation and differentiation, and humoral immune response. The Reactome enrichment results revealed that this module was mainly enriched in B cell activation and B cell receptor signaling pathways. Both GO and Reactome enriched terms indicated that genes in the steelblue module played critical roles in B cell-mediated immune responses. For the darkslateblue module, the genes were significantly enriched in GO terms of RNA catabolic and metabolic processes, RNA processing, translational initiation, viral gene expression and transcription, and cellular respiration, and were significantly enriched in Reactome terms of rRNA processing and translation, that implied genes in this module were mainly involved in the cellular metabolism. The genes in the salmon module were significantly enriched in GO terms of RNA localization, RNA transport and DNA biosynthetic process, and were significantly enriched in Reactome terms of SUMOylation, DNA damage response and DNA repair, that indicated genes in this module were mainly involved in regulating the cell cycle. The GO enrichment results revealed that genes in the blue module were significantly enriched in regulation of membrane potential, pattern specification process, G protein-coupled receptor (GPCR) signaling pathway and regulation of ion transmembrane transport. The Reactome enrichment results revealed that genes in this module were significantly enriched in GPCR ligand binding, peptide ligand-binding receptors and anti-inflammatory cytokines production pathways. Both functional enrichment analyses indicated that genes in the blue module were mainly involved in the cellular signal transduction pathways.

### 2.7. Host factors contribute to susceptibility of symptomatic influenza

**Figure 13:**
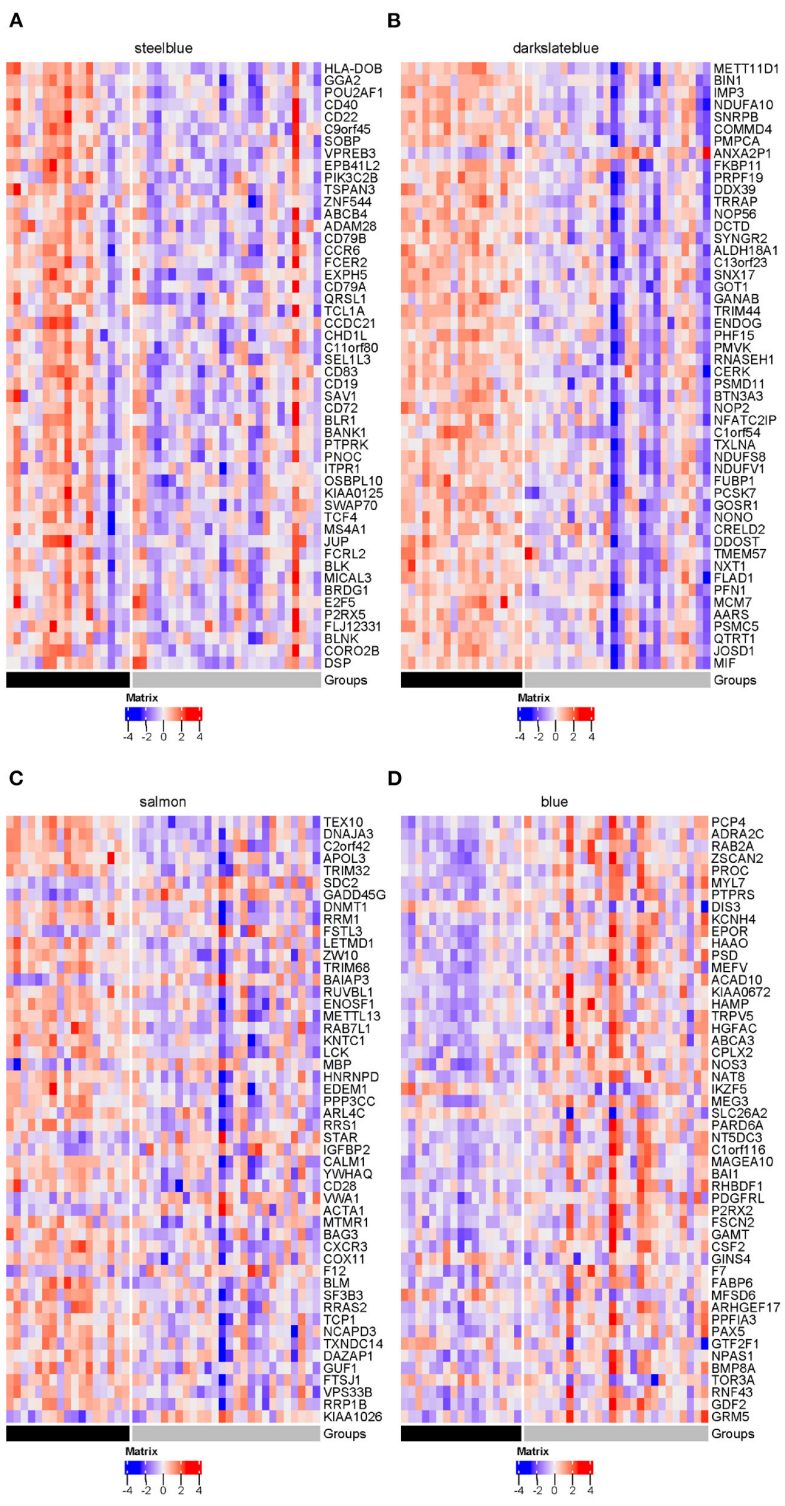
Heatmaps of DEGs in four disease modules (gray = SI; black = AU): **(A)** steelblue, **(B)** darkslateblue, **(C)** salmon and **(D)** blue. The top 50 genes with the smallest FDR-adjusted *p*-value of differential expression are presented in these heatmaps.

**Figure 14:**
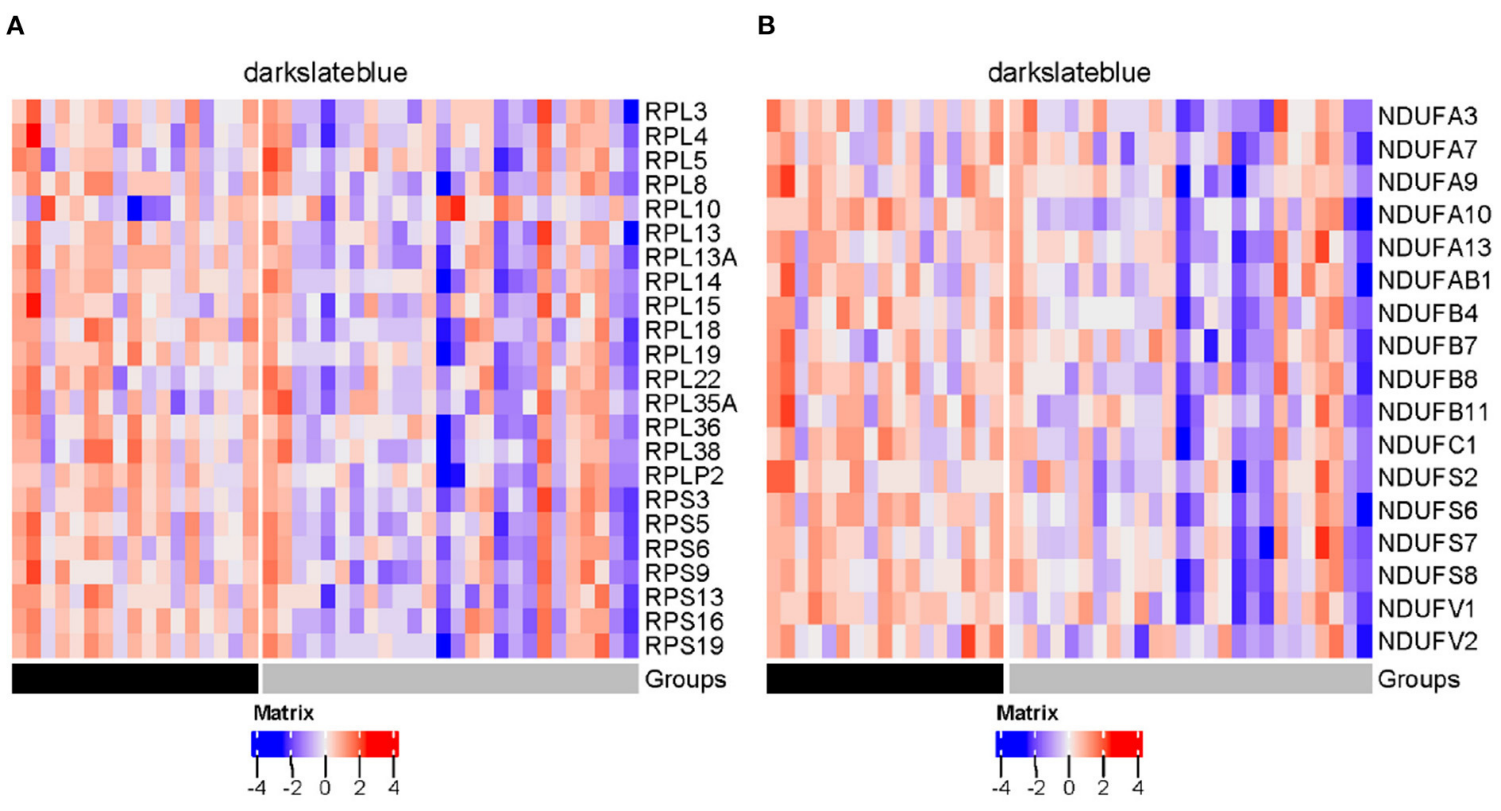
Heatmaps of genes encoding ribosomal proteins **(A)** and mitochondrial complex I subunits **(B)** in darkslateblue module (gray = SI; black = AU).

Influenza viruses depend on the host's cellular machinery to replicate, produce, and spread progeny virus particles. Further examination of the four disease modules identified a certain number of genes encoding host factors that could contribute to individual viral susceptibility. Of the three modules significantly down-regulated in the SI group compared to the AU group, the steelblue module showed the biggest decrease in modular expression, followed by the darkslateblue and salmon modules. Examination of differentially expressed genes (DEGs) within the steelblue module revealed a broad downregulation (Figure 13A). This downregulation included signaling components (*CD79A, CD79B, CD19, CD22, CD40, MS4A1, BLK*, and *BLNK*) involved in humoral immune response, driving B cell activation, proliferation, and differentiation. *CD79A* and *CD79B* are two important components of the B-cell receptor. *CD19* and *CD22* are typical B-cell activation markers. *POU2AF1* is a transcription factor which is vital for the response of B cells to antigens and required for the formation of germinal centers (Teitell, 2003). These results indicated that the DEGs within the steelblue module were typical B cell markers and transcription factors associated with B cell activation and differentiation. Downregulation of gene expression in the SI group was also observed in genes within the darkslateblue module (Figure 13B). This downregulation included genes involved in cellular metabolism, such as genes encoding the ribosomal proteins (Figure 14A) involved in viral gene expression and transcription, RNA catabolic process, translational initiation and protein targeting to ER, and genes encoding the mitochondrial complex I (NADH: ubiquinone oxidoreductase) subunits (Figure 14B) involved in cellular respiration, as well as genes *IMP3, NOP56, NOP2, QTRT1* involved in ncRNA and rRNA metabolic processes, ribonucleoprotein complex biogenesis, and ribosome biogenesis. For the salmon module involved in regulating cell cycle, the DEGs still displayed a broad downregulation in the SI group (Figure 13C). For the blue module involved in cellular signal transduction, the DEGs displayed an opposite trend of expression with an increased expression in the SI group (Figure 13D).

## 3. Discussion

In the present study, a systematic analysis of the relationship between influenza susceptibility and the baseline level of the NLR was developed. We examined five independent influenza challenge cohorts at the cellular level and found that individuals in the SI group had significantly higher baseline NLR than those in the AU group. The NLR achieved an AUC of 0.724 on the H3N2 data, and 0.736 on the external H1N1 data in predicting disease susceptibility to symptomatic influenza. The mechanisms underlying the association of the higher baseline NLR and the increased susceptibility to symptomatic influenza are poorly understood. The NLR is a biomarker conjugates two interconnected arms of the immune system: innate immunity and adaptive immunity. Neutrophils are the first line of innate immune defense against viral infection (Kaufmann, 2008). They migrate to infection sites for eliminating infectious particles, but also provide signals to other innate and adaptive immune cells about an invading foreign threat (Mantovani et al., 2011). The adaptive immunity is orchestrated mainly *via* T, B, and NK lymphocytes which provide antigen-specific responses. Prior studies revealed that the NLR was a particularly attractive measure of systemic inflammation (Zahorec, 2001). Neutrophils are crucial for innate immunity and are one of the main cell types involved in the inflammatory responses. The host innate immunity is activated by the inflammatory responses to control pathogen infection. Lymphocytes generate adaptive immune responses to eliminate specific pathogens. It is well established that the systemic inflammatory response is typically associated with decline in circulating lymphocyte count and increase in neutrophil count. Neutrophilia and lymphocytopenia are typical phenomena during systemic inflammation (de Jager et al., 2010; Templeton et al., 2014; Qun et al., 2020). Continuous infiltration of neutrophils at the site of infection raising an immune response produces exaggerated cytokines and chemokine that might result in the cytokine storm and contribute to severe disease during influenza virus infection (Bordon et al., 2013; Gu et al., 2019). Neutrophil extracellular traps (NETs) are released by neutrophils to contain infections. However, when not properly regulated, NETs have the potential to propagate inflammation (Porto and Stein, 2016; Twaddell et al., 2019). Beside this, more and more evidence has supported that neutrophils can significantly suppressed activation of CD4^+^ and CD8^+^ T cells, and further suppressed the immune responses (Pillay et al., 2013; Zemans, 2018). On the other side, lymphocytes are required for maintaining an effective immune response. The causes of lymphocytopenia as the marker of a depressed cell-mediated immunity, have been extensively studied (Cunha et al., 2011; Shen et al., 2014; Zhou and Ye, 2021). Lymphocytopenia also render the host susceptible to severe hyperinflammation (Chen et al., 2010). Thus, a healthy partnership between neutrophils and lymphocytes plays a very important role in the onset and resolution of inflammation which has a vital role in maintaining the health and integrity of an individual organism toward invading pathogens (Trammell and Toth, 2008; Buonacera et al., 2022). The condition of baseline NLR are indicative of the balance between the activation of host inflammatory responses and immune responses, therefore, it is a potential biomarker for predicting susceptibility to influenza virus infection. Hence, understanding of main NLR-related host factors associated with baseline systemic inflammatory status and influenza susceptibility may open doors for preventing influenza virus infection.

Despite many genomic and transcriptomic studies being conducted to identify host factors that are crucial for influenza susceptibility, the contribution of NLR-related host factors has not been fully explored. Using weighted gene co-expression network analysis (WGCNA), we identified four modules of the NLR-related systemic host factors associated with influenza susceptibility. In the discovery cohort for H3N2 influenza, we found that these four modules were not only significantly correlated with the baseline NLR, but also differentially expressed between the SI and AU groups. The reproducibility of these relationships was validated in an independent cohort for H1N1 influenza. Functional enrichment analyses revealed that these four modules were mainly involved in B cell-mediated immune responses, cellular metabolism, cell cycle, and cellular signal transduction, respectively.

Three of the four modules (i.e., modules involved in B cell-mediated immune responses, cellular metabolism, cell cycle) were significantly down-regulated in the SI group. Humoral immunity and cell-mediated immunity are two arms of adaptive immune responses. B cells play a major role in the humoral immune response (Marshall et al., 2018). Antigen binding to B cell receptor initiates B cell activation. The humoral immune system produces antigen-specific antibodies that can protect against primary and secondary infection. Antibodies against the hemagglutinin of influenza virus could prevent viral entry and replication (Wu and Wilson, 2020). Beside this, rapid B cell responses contribute to efficient viral clearance through neutralizing the virus and reducing virus spread (Gerhard et al., 1997; Rothaeusler and Baumgarth, 2010). The overall immune status at baseline, including the composition of B cell subsets and the up- or down-expression of genes related to B cell receptor signaling have been found to predict post-vaccination responses (Tsang et al., 2014; Fourati et al., 2016; HIPC-CHI Signatures Project Team and HIPC-I Consortium, 2017; Parvandeh et al., 2019). These studies implicated the pre-vaccination status of B cell signaling as important indicators of immune state that influenced the antibody response as well as vaccination outcome. Our study identified several B cell signaling pathways and transcription factors (*POU2AF1* and *E2F5*) that regulated B cell activation and differentiation were significantly down-regulated in the SI group compared to the AU group. These results indicated the status of B cell signaling at baseline can be a useful predictor of influenza symptomatic infection. Moreover, influenza virus infection usually reprograms host cell's metabolism to assist virus replication. Influenza viruses require host cell ribosomes for expression of viral proteins. Ribosomal proteins (RPs) are major components of ribosomes. Recent studies revealed that RPs possessed antiviral function. Some RPs can interact with viral proteins to inhibit virus transcription (Abbas et al., 2012; Li et al., 2016). The ribosomal protein *RPL10* was identified as a downstream effector of the NIK (NSP-interacting kinase)-mediated antiviral signaling pathway (Rocha et al., 2008). Beside this, the ribosomal protein *RPL13A* was reported as an innate immune factor for antiviral defense (Mazumder et al., 2014). Previous work reported that influenza virus suppressed host cellular respiration which was related to mitochondrial dysfunction (Derakhshan et al., 2006), but the molecular mechanism by which influenza virus alters cellular respiration is still unclear. Viruses deeply rely on host post-translational modifications for their replication. SUMOylation is an important post-translational modification controlling various cellular processes. Viruses could take advantage of the cellular SUMOylation system to facilitate viral propagation (Pal et al., 2011; Han et al., 2014; Domingues et al., 2015), but the SUMOylation system could also serve an antiviral function to restrict viral replication. Recent studies have indicated SUMOylation with a critical role in activating host intracellular pathogen defenses (Boutell et al., 2011; Li et al., 2012). Specifically, the SUMO pathway was revealed to contribute to intrinsic antiviral resistance to herpes simplex virus type-1 infection (Boutell et al., 2011). Moreover, influenza viruses introduced DNA damage in host cells during infection (Li et al., 2015). The DNA damage response (DDR) is a complex signal transduction pathway that can detect DNA damage and transduce this information to the cell to influence cellular responses to DNA damage (Ciccia and Elledge, 2010). Prior studies revealed that the DDR may inhibit (Lau et al., 2004) viral replication.

We further identified the module involved in cellular signal transduction was significantly up-regulated in the SI group, and eigengenes of this module were positively correlated with the baseline NLR. Within this module, we identified several key signaling pathways contributing to the efficiency of viral replication, including GPCR signaling and ion transport pathways. Influenza virus infection induces activation of a variety of cellular signaling pathways, which are required by virus replication (Ludwig et al., 2006; Ludwig, 2009). GPCRs contribute directly to stimulate the *Raf/MEK/ERK* signaling pathway (Rozengurt, 2007), which is crucial for influenza virus replication and has been demonstrated to possess antiviral properties (Pleschka et al., 2001). Ion channels expressed by host cells have emerged as key regulators of virus entry, and ion channels drugs have attracted some attention as suitable antiviral agents. Our findings highlighted the potential contribution of some key pathways involved in B cell-mediated immune responses, cellular metabolism, cell cycle, and signal transduction to influenza susceptibility. These identified pathways were concordant with underlying mechanisms that had previously been reported to be associated with host-virus interactions. The WGCNA network analysis is an unsupervised approach, which does not use a priori phenotype information (e.g., infection susceptibility). Thus, it provided an integrated and global view of host factors and allowed us to gain insights into the main host factors contributing to a healthy partnership between neutrophils and lymphocytes, and identify the main NLR-related host factors associated with influenza susceptibility.

The recent coronavirus disease 2019 (COVID 19) pandemic has resulted in significant morbidity and mortality worldwide (World Health Organization, 2020). Influenza and COVID-19 are both contagious respiratory illnesses, but COVID-19 is caused by infection with a coronavirus first identified in 2019. The NLR has recently generated a lot of interest regarding the role of potential poor prognosis in COVID-19 patients. Many studies have shown that the NLR was associated with disease severity and mortality for COVID-19 patients (Chan and Rout, 2020; Kong et al., 2020; Lagunas-Rangel, 2020; Regolo et al., 2022). A number of recently published studies have found that an elevated NLR on admission can serve as an early warning signal of severe COVID-19 (Feng et al., 2020; Liu J. et al., 2020; Liu Y. et al., 2020). A recent metanalysis (Henry et al., 2020) showed that lymphopenia and neutrophilia at hospital admission are associated with poor outcomes in patients with COVID-19. These studies exhibited that systemic inflammation played a key role in the development of severe COVID-19 which is in concordance with influenza. To our knowledge, no studies on baseline (i.e., prior to exposure to SARS-CoV-2) NLR to COVID-19 susceptibility have been reported. Influenza is our best comparative model for COVID-19 (Moore et al., 2020), hence our work can serve as a comparative model to provide insights into the COVID-19 susceptibility.

Several limitations of this study are noteworthy. The number of baseline samples available in the influenza challenge studies were low. We only included symptomatic infected and asymptomatic uninfected subjects in our study, thus the findings reported here are restricted to individuals who unambiguously reported health or illness, i.e., viral titer and symptom status agree. Moreover, individuals participated in the challenge studies were young and healthy adults, which may limit the broad applicability of our results to children, elderly or high-risk populations. Furthermore, as this study focused on H3N2 and H1N1 influenza, the association between baseline NLR and influenza susceptibility may not be extended to other type of influenza strains and infections.

In conclusion, our work identified the NLR as a simple and useful biomarker for predicting disease susceptibility to symptomatic influenza. An elevated NLR was detected in susceptible hosts, who may have defects in B cell-mediated immunity or impaired function in cellular metabolism, cell cycle, or signal transduction. The understanding of main NLR-related host factors associated with baseline systemic inflammatory status and influenza susceptibility may open doors for preventing influenza virus infection. Further study will be required to understand the underlying mechanism of susceptibility to influenza virus infection, and may yield therapeutic targets.

## 4. Materials and methods

### 4.1. Data availability

For the GSE73072 data set, we directly downloaded its preprocessed expression matrices from GEO, which had been normalized using the robust multi-array (RMA) method and log2-transformed. For the GSE61754 data set, we utilized the expression matrix available from GEO, which had been preprocessed using the variance stabilization and normalization method. For the GSE111368 cohort, we obtained the expression profiles from GEO which had been log2-transformed and normalized with a 75th percentile-shift algorithm by the original author. The infection and symptom status for the GSE73072 cohort were found at the web link (https://drive.google.com/open?id=0B2vLBS4X1c1ENzZpT216eGY1RjQ) provided by the original author, and those for the GSE61754 and GSE111368 cohorts were retrieved from their GEO Series Matrix Files.

### 4.2. Deconvolving whole blood gene expression samples

To quantify the proportions of human blood cell types, we utilized a signature matrix, named sigmatrixMicro provided in a previous study (Monaco et al., 2019), whose transcriptomic profiles were generated by microarray. The sigmatrixMicro consisted of 819 cell type-specific genes in 11 immune cell types. We next performed deconvolution with support vector regression using the CIBERSORTx method (Newman et al., 2019). Cell proportions within whole blood samples were estimated by combining CIBERSORTx with sigmatrixMicro in non-log linear space. All microarray data sets were quantile normalized before running CIBERSORTx. Bulk-mode batch correction was applied to remove technical differences between whole blood mixtures and the signature matrix.

To demonstrate that the differences in estimated baseline NLR between the SI and AU groups were robust to the signature matrix used, we quantified the proportions of human blood cell types in the same manner as described above except that two different signature matrices (LM22 and immunoStates) were used instead of sigmatrixMicro. The LM22 signature matrix contains 547 genes in 22 immune cell types and was obtained from the CIBERSORT website (https://cibersort.stanford.edu; Newman et al., 2015). It was built using samples from healthy subjects and profiled by Affymetrix microarray. The immunoStates provided in a previous study (Vallania et al., 2018) consisted of 317 cell type-specific genes in 20 immune cell types and was built using 6,160 samples with different disease states across 42 microarray platforms.

### 4.3. Application of batch correction to GSE73072 cohorts

The discovery cohort (GSE73072 H3N2) included two challenge studies. Principal component analysis (PCA) revealed that the discovery cohort included two sample batches, therefore the *removeBatchEffect* function provided in the limma package was used to correct the batch effects of gene expression values in the discovery cohort (Figure 1A). The PCA analysis was performed again on the corrected data, and the batch effects of the two challenge studies in the discovery cohort were basically eliminated. The same strategy was employed to remove batch effects in the GSE73072 H1N1 cohort (Figure 1B).

### 4.4. Co-expression network construction by WGCNA

WGCNA is the most widely used approach for weighted correlation network analysis. Co-expression networks were built using the R package WGCNA. The analysis was performed using the batch corrected gene expression profiles and only genes with variances ranked in top 9,000 were used. The soft-threshold power β was set to 10 which was selected based on the *pickSoftThreshold* function. A hierarchical clustering was performed using 1-TOM with β = 10 as the pairwise distance and average linkage distance as the cluster distance. We identified modules using dynamic tree cut approach with a minimal module size of 30 and a deepsplit cutoff of 4, and those closely related modules whose correlations of module eigengenes larger than 0.75 were merged by setting a branch merge cutoff height of 0.25.

### 4.5. Functional enrichment analysis

The pathway enrichment analyses of genes in four disease modules were analyzed. The biological processes from gene ontology (GO) and molecular pathways from the Reactome database are two of the most commonly used pathway enrichment analysis resources. GO enrichment analyses were conducted with *enrichGO* function in the R package clusterProfiler (version 3.18.1) with FDR < 0.05, and here the background set of genes was defined as default. Reactome enrichment analyses were performed with *enrichPathway* function in the R package ReactomePA (version 1.9.4).

### 4.6. Meta analysis and statistical analysis

Meta-analyses using random-effect models were performed with *metagen* function in the R package meta. Hedges' adjusted *g* (Cooper et al., 2019) was used to standardize the mean difference in the NLR between the SI and AU groups. The pooling weights were calculated as inverse of the effect size variance. All tests were performed two-sided, and a *p*-value cutoff for statistical significance was set as 0.05.

Wilcoxon tests were conducted to identify statistically significant differences in estimated baseline NLR, lymphocyte, and neutrophil proportions between the SI and AU groups. Statistically significant changes in modular expression between the SI and AU groups were assessed using Student's *t*-test. Statistically significances were indicated as follows: ns, not significant; ^*^p < 0.05, ^**^p < 0.01, ^***^p < 0.001, ^****^p < 0.0001.

## Data availability statement

The datasets presented in this study can be found in online repositories. The names of the repository/repositories and accession number(s) can be found in the article/supplementary material.

## Ethics statement

Ethical review and approval was not required for the study on human participants in accordance with the local legislation and institutional requirements. Written informed consent for participation was not required for this study in accordance with the national legislation and the institutional requirements.

## Author contributions

Conceptualization, writing—original draft preparation, supervision, project administration, and funding acquisition: WS. Methodology, validation, formal analysis, and visualization: GW and WS. Software and writing—review and editing: GW, CLv, CLi, and WS. Data curation: GW, CLv, and WS. All authors have read and agreed to the published version of the manuscript.

## Funding

This work was supported by Shantou University Medical College Development Funds (510858027).

## Conflict of interest

The authors declare that the research was conducted in the absence of any commercial or financial relationships that could be construed as a potential conflict of interest.

## Publisher's note

All claims expressed in this article are solely those of the authors and do not necessarily represent those of their affiliated organizations, or those of the publisher, the editors and the reviewers. Any product that may be evaluated in this article, or claim that may be made by its manufacturer, is not guaranteed or endorsed by the publisher.
